# Temperature effects on prey and basal resources exceed that of predators in an experimental community

**DOI:** 10.1002/ece3.4695

**Published:** 2018-11-26

**Authors:** Madhav P. Thakur, John N. Griffin, Tom Künne, Susanne Dunker, Andrea Fanesi, Nico Eisenhauer

**Affiliations:** ^1^ German Centre for Integrative Biodiversity Research (iDiv) Halle‐Jena‐Leipzig Leipzig Germany; ^2^ Institute of Biology Leipzig University Leipzig Germany; ^3^ Netherlands Institute of Ecology (NIOO‐KNAW) Wageningen The Netherlands; ^4^ Department of Biosciences Swansea University Swansea UK; ^5^ Helmholtz Centre for Environmental Research—UFZ Leipzig Germany

**Keywords:** ectotherms, litter decomposition, microbial biomass, multiple predator effects, trait‐mediated interactions, trophic cascade, trophic mismatch

## Abstract

Climate warming alters the structure of ecological communities by modifying species interactions at different trophic levels. Yet, the consequences of warming‐led modifications in biotic interactions at higher trophic levels on lower trophic groups are lesser known. Here, we test the effects of multiple predator species on prey population size and traits and subsequent effects on basal resources along an experimental temperature gradient (12–15°C, 17–20°C, and 22–25°C). We experimentally assembled food web modules with two congeneric predatory mites (*Hypoaspis miles* and *Hypoaspis aculeifer*) and two Collembola prey species (*Folsomia candida* and *Proisotoma minuta*) on a litter and yeast mixture as the basal resources. We hypothesized that warming would modify interactions within and between predator species, and that these alterations would cascade to basal resources via changes in the density and traits (body size and lipid: protein ratio) of the prey species. The presence of congeners constrained the growth of the predatory species independent of warming despite warming increased predator density in their respective monocultures. We found that warming effects on both prey and basal resources were greater than the effects of predator communities. Our results further showed opposite effects of warming on predator (increase) and prey densities (decrease), indicating a warming‐induced trophic mismatch, which are likely to alter food web structures. We highlight that warmer environments can restructure food webs by its direct effects on lower trophic groups even without modifying top‐down effects.

## 1 INTRODUCTION

Climate warming can modify the structure of ecological communities by altering biotic interactions (Dillon, Wang, & Huey, 2010; Gilbert et al., 2014; Gilman, Urban, Tewksbury, Gilchrist, & Holt, 2010; Walther et al., 2002). For instance, higher temperature can amplify both intra‐ and interspecific competition by increasing individuals’ consumption rates owing to greater metabolic demands in ectotherms (Amarasekare & Coutinho, 2014; Lang, Rall, & Brose, 2012; Reuman, Holt, & Yvon‐Durocher, 2013). If these shifts in competitive dynamics, for instance among species at higher trophic levels (e.g., predators), affect their prey community and thereby the basal resources (i.e., through trophic cascades), we may expect shifts in the structure of ecological communities (Gilman et al., 2010). Previous studies have outlined that the strength of trophic cascades becomes weaker when predators suffer from greater negative interactions (Finke & Denno, 2004), such as in the form of interference competition or intraguild predation (Schmitz, 2007). In contrast, the strength of trophic cascades may magnify if greater consumption rates of predators at higher temperature remain unconstrained in relation to their negative interactions (Kratina, Greig, Thompson, Carvalho‐Pereira, & Shurin, 2012; Svensson et al., 2017).

Greater competition among predators should occur at higher temperatures when their foraging demands (e.g., attack rates) are maximal (Amarasekare, 2015). These competitive interactions may take place in the form of exploitative (Milazzo, Mirto, Domenici, Gristina, & Genner, 2013) and/or interference competition (Lang et al., 2012). Exploitative competition results from the competition among the consumers for shared and limited resources (Amarasekare, 2002; Case & Gilpin, 1974; Holt, Grover, & Tilman, 1994; Schoener, 1983). Importantly, exploitative competition at higher temperature may increase when there is a mismatch between the metabolic demands of the predators and the supply rates or the availability of prey at that temperature level. Warming may also increase interference competition among predators by increasing their movements and encounters with conspecifics and/or the individuals from competing predator species (Lang et al., 2012). This form of competition can arise independent of resource availability (Schoener, 1983) and mainly occurs due to territoriality and direct combat between the predators (Amarasekare, 2002; Schmitz, 2007; Schoener, 1983).

The results of intra‐ and interspecific competition in either exploitative or interference forms may trigger a decline in predators’ ability to consume resources and thus a decline in their population (Amarasekare, 2002). Alternatively, predators may also avoid competition either by greater resource partitioning at higher temperature or via the ability of predators to express plasticity in thermal tolerance to optimize their metabolic demands (Gunderson & Leal, 2016). Besides competitive interactions, some studies have also pointed out that higher temperature can lead to intraguild predation when predator species have a greater spatial overlap of their habitat (Barton & Schmitz, 2009; Schmitz, 2007). Importantly, if temperature changes the relative effects of intra‐ and interspecific interactions among predators, it would potentially also influence the effects of multiple predators on the prey community and further the resource of the prey community (Schmitz, 2007; Sih, Englund, & Wooster, 1998).

The contemporary food web literature consists of two potential mechanisms for linking predation effects on lower trophic groups (i.e., trophic cascade) in a given environment (Schmitz, Krivan, & Ovadia, 2004). These two mechanisms relate to how predators influence prey density and/or prey traits. The direct consumption of prey by their predators lowers prey density and consequently enhances the amount of the resources for prey. Trophic cascades via shifts in prey traits are usually indirect effects of predators via their nonconsumptive effects on their prey (Schmitz et al., 2004; Trussell, Matassa, & Ewanchuk, 2017)—also called nonlethal effects of predators (Peacor & Werner, 2001). However, lethal effects of predators (via direct encounter between predator and prey) can also trigger shifts in the traits of surviving prey, such as changes in their physiological and behavioral traits, which may affect their consumption patterns, thereby altering the amount of their resources (Schmitz, 2017). Such density‐ and trait‐mediated interactions can thus be affected by the interactions between multiple predators (Steffan & Snyder, 2010).

Higher temperature effects on the lower trophic groups are, however, not limited to operate via predatory interactions only. Temperature effects can simultaneously influence both prey and their resources also by augmenting their metabolism (Antiqueira, Petchey, & Romero, 2018). In fact, predator‐induced regulation of prey and their resources are likely to disappear if temperature eliminates predator species because of their greater vulnerability (e.g., due to higher metabolic demands than their prey) at higher temperature (Petchey, Mcphearson, Casey, & Morin, 1999; Thakur, Kunne, Griffin, & Eisenhauer, 2017; Zarnetske, Skelly, & Urban, 2012).

Here, we investigate how temperature alters intra‐ and interspecific predator interactions and their implications for trophic cascades (indirect effects on basal resources) using experimental communities. We ask two main questions: (a) Does higher temperature affect interspecific interactions relative to intraspecific interactions among the predators? (b) Do predators and temperature interactively alter prey populations and the basal resource? To answer these two questions, we assembled experimental food webs with two congener microarthropod predator species and a prey community of litter microarthropod detritivores across a temperature gradient and measured changes in the predator population, prey population, and prey traits. We assessed body length (morphological trait) and lipid to protein ratio of unconsumed prey individuals (physiological trait) to investigate the strength of trait‐mediated effects. These traits are fundamentally linked to organisms’ physiological adjustments at varying temperature and stress environments, such as in the presence of predators (Clarke, 2017; Gardner, Peters, Kearney, Joseph, & Heinsohn, 2011; Hochachka & Somero, 1973; Karasov & Martinez del Rio, 2007). In order to quantify trophic cascades, we measured two basal resources (microbial biomass and litter biomass) in our experimental communities. We hypothesize that warming would enhance competition within and between predators, which are likely to weaken trophic cascades due to potential decline in predators.

## 2 MATERIALS AND METHODS

### 2.1 Experimental community

Our experimental community consisted of two predatory mite species as predators and two Collembola species as their prey. The two congeneric predatory species were *Hypoaspis aculeifer* and *Hypoaspis miles*. The two prey species were *Folsomia candida* and *Proisotoma minuta,* both belonging to the Isotomidae family. These Collembola species were cultured in the laboratory facility of Leipzig University (cultured with dry yeast at a temperature of 14°C), whereas predatory mites were commercially obtained from *Schneckenprofi* in Germany. These organisms occur mostly in the litter and top layers of the soil. Collembola are generally fungal grazers, but also ingest litter material in their diet, and thus are important litter detritivore species (Chahartaghi, Langel, Scheu, & Ruess, 2005). The Collembola species *F. candida* is larger in body size (body length ranging from 1,500 to 3,000 µm) than *P. minuta* (body length ranging from 600 to 1,100 µm) (Thakur, Künne, Griffin, & Eisenhauer, ). The two predatory species range from 700 to 800 µm in their body sizes (Jess & Bingham, 2004), but despite their smaller body size, they both are known as voracious predators of Collembola species (Heckmann, Ruf, Nienstedt, & Krogh, 2007), as confirmed by our own trials. However, their foraging success may vary with temperature (Thakur, Künne, et al., ). For instance, *H. miles* were found to consistently suppress both prey species, whereas *H. aculeifer* had greater success in suppressing the smaller prey (*P. minuta*) at higher temperatures (Thakur, Künne, et al., ). Importantly, our experimental communities present a particular scenario of predators being relatively smaller in body size than their prey (specifically the larger prey species: *F. candida)*. Greater vulnerability of predators than their prey at higher temperatures often relates to their larger body size (Petchey et al., 1999; Vucic‐Pestic, Ehnes, Rall, & Brose, 2011). This is due to greater metabolic costs than energetic gains in predators when exposed to higher temperature (Iles, 2014; Lemoine & Burkepile, 2012). The same could be assumed for prey species that are larger in body size than their predators based on metabolic principles (Brown, Gillooly, Allen, Savage, & West, 2004).

Despite the body size differences between the two prey species, their thermal performances match to some extent. For instance, both prey species have been reported to hatch eggs in the temperature range of 15–23°C, which usually takes 6–10 days (Fountain & Hopkin, 2005; Park, 2007). However, our previous study with similar substrates that we used in this study showed that the thermal plasticity (change in mean body length) was higher in *F. candida* populations than in *P. minuta* when they were grown as monocultures (Thakur, Künne, et al., ). Without predators, both prey species showed similar population trajectories in their monocultures, but *P. minuta* declined as temperature exceeded 20°C (Thakur, Künne, et al., ). In favorable environmental conditions (e.g., sufficient moisture), their generation time (from egg to reproductive stage) is about two to three weeks (Fountain & Hopkin, 2005; Park, 2007).

As we used two closely related predators (i.e., congeners) in this experiment, we assumed that their resources will overlap and hence that they will compete for resources. At ambient temperature (the temperature at which we had cultured the prey species), both predators were shown to suppress both prey species (Thakur, Künne, et al., ). At 15°C, the generation time of *H. miles* usually is between 30 and 35 days, which could shorten to as low as to 12 days at 24°C (Wright & Chambers, 1994). The generation time of *H. aculeifer* is slightly higher than that of *H. miles* at higher temperatures. For example, Lobbes and Schotten (1980) reported about 20 days of generation time for *H. aculeifer* when cultured at 24.5°C. However, the same authors reported relatively shorter generation time of *H. aculeifer* at 15°C (~22 days) than of *H. miles*. These generation times are, however, likely to vary with the quality of food resources.

### 2.2 Experimental design

We performed a microcosm experiment with the above‐described experimental community along a temperature gradient. We used temperature treatments with a day and night cycle of 16 hr and 8 hr, respectively, but with no light to keep dark conditions for animals. The dark conditions for litter and soil animals resemble more to their natural habitat conditions. The experiment ran in three different temperature regimes: 12–15°C (12°C for 8 hr night and 15°C for 16 hr day; representing ambient conditions that the two Collembola species had experienced for several generations; Thakur, Künne, et al., ), 17–20°C (17°C for 8 hr night and 20°C for 16 hr day), and 22–25°C (22°C for 8 hr night and 25°C for 16 hr day). The warmer temperature regimes (+5 and +10°C) were to mimic moderate to extreme warming scenarios for the next 100 years as per the predictions of the IPCC for several regions (Buckley & Huey, 2016; IPCC, 2014). Our previous trials with the monocultures of the model species showed that all these species survived in these temperature regimes (Thakur, Künne, et al., ).

In total, we established four communities (two prey species, two prey species + predator 1, two prey species + predator 2, and two prey species + predator 1 + predator 2) across three temperature regimes (12–15°C, 17–20°C, and 22–25°C), each replicated five times. At the start of the experiment, we added 10 individuals of each Collembola species. These individuals were carefully sorted from the laboratory cultures to be identical in their body size. Immediately after the addition of Collembola individuals (within hours), we added predatory mites in the following combination: predator monocultures received six individuals, whereas predator polycuture treatments received three individuals of each predator species. By keeping the total predator density constant, we established a substitutive design. This design tests the effect of multiple predators, while holding total predator density constant; it thus tests whether interspecific interactions differ from intraspecific interactions, that is, the substitutability of predator species (Schmitz, 2007). Under this design, the combined species effects should be the same as their average in monoculture (Schmitz, 2007). While this is the most commonly used design in studies addressing multiple predator effects in food webs (Griffin, Byrnes, & Cardinale, 2013), it should be kept in mind that it does not allow to clearly differentiate intraspecific from interspecific interactions. Although the starting density of predators in mixed community was lower (i.e., three individuals each), our previous experiments have shown that such a lower density is still sufficient to establish a higher population over the experimental period (Thakur et al., 2015; Thakur, van Groenigen, Kuiper, & De Deyn, 2014) compared to this study.

The microcosms used in this study were ventilated (allowing air flow) petri dishes (14 cm in diameter) with litter material (sterilized twice, C:N ratio ~17) mixed with dry yeast as the main substrate for Collembola species. We added 1.5 g dry weight of litter material in each petri dish with 10 mg of dry yeast dissolved in 1 ml of deionized water. In this way, we stimulated fungal growth on litter before the animal communities were established. We used a layer of two filter papers on the bottom of petri dishes for maintaining a well‐distributed moisture (Supporting Information Figure S1).

The experiment ran in reach‐in growth chambers with the above‐mentioned three temperature regimes. For each temperature regime, we used two growth chambers (CLF Plant Climatics GmbH). For the entire duration of the experiment, we maintained a relative humidity of 70%. We added 1 ml of deionized water every day in the first week of the experiment to enhance the fungal growth on litter material. From the second week on, after fungal growth was visually observed, we added the same amount of deionized water every third day until the end of the experiment. The experiment ran for 60 days after the addition of animals.

### 2.3 Experimental harvest and response variables

At the end of the experiment, we extracted animals from all the microcosms and determined microbial and litter biomass. Our response variables can be divided into five main components: (a) predator density, (b) prey density, (c) prey traits, (d) microbial biomass on litter material, and (e) litter mass loss (dry biomass at the start of the experiment—dry biomass at the end of the experiment).

Predator and prey species were extracted from the petri dish using a heat extraction technique (with a gradual heating of +5°C per day for six days starting from 25°C up to 55°C; Macfadyen, 1961). Animals were first collected in glycol water solution (1:1 ratio) and then transferred to ethanol (70%) for counting and trait measurements. Predatory mites and Collembola species were counted using a dissecting microscope.

We determined two traits of the prey species: body length, and lipid and protein content of the prey individuals. However, these measurements were possible only for one of the prey species—*F. candida*. We did not recover a sufficient density of the other prey species (*P. minuta*; at least 10 individuals) required to make trait measurements in most of the treatments with warming and predators (Supporting Information Figure S2). Hence, our trait results are based on the measurement of *F. candida* only. We randomly selected 10 individuals and used an inverted microscope (Leica DMI 4000B) at 40× magnification to measure the body length of *F. candida* individuals. Lipid and protein measurements of *F. candida* individuals were carried out using the FT‐IR spectroscopic method (Wagner, Liu, Langner, Stehfest, & Wilhelm, 2010). About 20 individuals of *F. candida* were collected from ethanol (stored about one month) and crushed into a paste for the lipid and protein measurements in a volume of 10 µl. The ethanol fraction of the sample was evaporated and suspended in distilled water. Three times 2 µl of this suspension per sample were spotted on a 384 silicon well plate (Bruker Optics, Ettlingen, Germany) and dried at 40°C for at least 10 min. Samples were measured with a Bruker Vector 22 Laser‐unit, coupled with a HTS‐XT microtiter module (Bruker Optics, Karlsruhe, Germany; Wagner et al., 2010). In the range of 4,000–700 cm^−1^, 64 scans were performed. Spectra were analyzed with the software OPUS (v5.0, Bruker Optics, Germany), meaning that technical replicates were averaged; a baseline correction and a correction of CO_2_‐bands in the range between 2,400–2,200 cm^−1^ were performed.

At the end of the experiment, a fraction of fresh litter material (~0.25 g) was further collected from the microcosms for the measurement of microbial biomass C. We used the substrate‐induced respiration technique to determine litter microbial biomass C (Scheu, 1992). We added 0.016 g of D‐glucose on the fresh litter, and the subsequent respiration was measured every hour for at least 15 hr at 20°C. The average of the lowest three readings within the first 10 hr was taken as the “maximum initial respiratory response” (MIRR, µg O_2_ hr^−1^ g soil dw^−1^). Microbial biomass (mg C g^−1^) was calculated as 38 × MIRR (Beck et al., 1997). Finally, we collected all the litter used for animal extraction as well as for microbial biomass assessment and air‐dried them at 70°C for 48 hr. The dry biomass of litter was assessed, and litter biomass loss was calculated from subtracting it from the initial dry litter biomass (i.e., 1.5 g).

### 2.4 Statistical analyses

We applied linear models to test the interactive effects of predator communities (as a categorical variable: none as a control, *H. aculeifer* monocultures, *H. miles* monocultures, and both predators together) and temperature (as a categorical variable with three levels) on prey responses as well as on microbial biomass C and litter biomass loss. As we lacked adequate replicates of the prey trait data from predator monoculture treatments at the highest temperature, we opted to use predator presence versus absence in analyzing prey trait responses instead of using four levels of predator treatments. For the predator interaction effect on predator‐specific densities across different temperature regimes, we ran linear models with only three levels of the predator treatment without the predator control treatment. All response variables were analyzed using Gaussian error terms, except for the count data. There were two response variables as count data in our analyses: prey density and predator density. For predator density responses, we found an overdispersion in the regression model with Poisson error terms. Thus, we regressed predator density against treatments using negative binomial error terms, which resolved overdispersion in the model. For prey density, we found both overdispersion and zero inflation (Supporting Information Figure S3) when modeled with Poisson as well as with negative binomial error terms. We thus used zero inflation models with negative binomial error terms. All linear regression model assumptions were met (e.g., homogeneity of variance). We also carried out post‐hoc Tukey tests on mixed models. For all our regression models (except the zero‐inflated models), we also report the measure of effect size for treatments by using partial omega‐squared (*⍵* ^2^_partial_) (Olejnik & Algina, 2003).

We used a path model to assess the density‐ and trait‐mediated indirect effects of predators (presence vs. absence) and warming on trophic cascades. As trait data were only available for *F. candida,* we also only included the density data of *F. candida* in our path model. Density‐ and trait‐mediated effects on basal resources were calculated as the indirect effects from the path model. The total indirect effects were calculated as the product of standardized path coefficients and summed over each path along a given basal resource (Shipley, 2016). For example, the total indirect effect of temperature on microbial biomass was calculated as: Σ[(standardized path coefficient of temperature to prey density × standardized path coefficient of prey density to microbial biomass), (standardized path coefficient of temperature to prey body length × standardized path coefficient of prey body length to microbial biomass), (standardized path coefficient of temperature to prey lipid: protein ratio × standardized path coefficient of prey lipid: protein ratio to microbial biomass)]. Please note though that our experimental set‐up does not allow to separate consumptive and nonconsumptive effects of predators on prey traits as often done in experiments to estimate trait‐mediated effects by preventing direct encounters between predators and prey (Schmitz et al., 2004; Trussell et al., 2017). Hence, these indirect effects of predators on basal resources are the result of both consumptive and nonconsumptive effects of predators on prey species and should be interpreted with caution.

We also included direct paths from temperature and predator presence on the two basal resources to account for potential effects that may not be explained by our prey measurements (indirect paths). For the convenience in the interpretation of coefficients, we used predator communities and temperature as linear terms in our path model. We further used this approach as the majority of our regression results (Table 1) remained unchanged when treatments were used as continuous predictors (Supporting Information Table S1).

**Table 1:** Results of linear mixed models with temperature and predator community effects on predator and prey densities, prey traits, and basal resources. Please note that predator community effects on the predator response have only two levels of predator treatment (monocultures and polyculture), whereas for the prey density and two basal resources, the predator treatment has four levels (none, *Hypoaspis aculeifer* monoculture, *H. miles* monoculture, and both predators together). For two prey traits, we could only use predator absence and presence in our statistical models (see methods for the explanation). The statistical significance (*F*‐ and *p*‐values) of the response variables was determined by *F*‐tests. *p*‐values lower than 0.05 are statistically significant and indicated in bold. *df* stands for degrees of freedom. The measure of effect size is given by partial omega‐squared (*⍵* ^2^_partial_)

	Predator communities (*P*)			Temperature (*T*)			*P* × *T*		
	*F*‐value*_df_*	*p*‐value	*⍵* ^2^_partial_	*F*‐value*_df_*	*p*‐value	*⍵* ^2^_partial_	*F*‐value*_df_*	*p*‐value	*⍵* ^2^_partial_
Predator response									
*H. aculeifer* density	**11.68_1,24_**	**<0.01**	**0.12**	**5.19_2,24_**	**0.01**	**0.05**	0.74_2,24_	0.48	<0.01
*H. miles density*	**21.90_1,24_**	**<0.001**	**0.34**	**5.35_2,24_**	**0.01**	**0.16**	2.18_2,24_	0.13	0.13
Prey response									
Prey density	1.76_3,35_	0.17	—	**4.55_2,35_**	**0.01**	—	1.95_6,35_	0.09	—
Body size	<0.01_1,32_	0.93	0.02	**38.58_2,32_**	**<0.001**	**0.66**	**6.09_2,32_**	**<0.01**	**0.21**
Lipid: protein ratio	**17.55_1,29_**	**<0.001**	**0.32**	2.15_2,29_	0.13	0.06	0.08_2,29_	0.91	0.05
Basal resource response									
Microbial biomass C (log‐scaled)	0.74_3,46_	0.53	0.01	0.15_2,46_	0.85	0.03	0.64_6,46_	0.69	0.03
Litter mass loss	0.15_3,48_	0.92	0.04	**29.29_2,48_**	**<0.001**	**0.48**	0.70_6,48_	0.64	0.03

All the model structures were identical to our main regression models except that we used negative binomial models for prey density, which was log‐transformed to reduce overdispersion (although still present). This was due to computational constraints in incorporating zero‐inflated negative binomial models in path models. Please note though that the results of log‐transformed prey density with negative binomial error resemble with that of zero‐inflated negative binomial models (Table 1 and Supporting Information Table S2). We used Shipley's test of d‐separation which yields Fisher's C statistic (Chi‐square distributed) for the assessment of the overall fit of our path model (Shipley, 2009).

All statistical analyses were carried out in R statistical software version 3.2.5 (R Core Team, 2016). Linear model diagnostics (e.g., test of overdispersion and homogeneity of variance) were performed in the DHARMa package for R (Hartig, 2017). The zero inflation tests for the count data were also performed in the DHARMa package (Hartig, 2017). The negative binomial zero‐inflated model was run in the pscl package (Zeileis, Kleiber, & Jackman, 2008). We carried out *F*‐tests on linear models using the car package for R (Fox & Weisberg, 2011). Post‐hoc tests were performed used the multcomp package (Hothorn, Bretz, & Westfall, 2008) and the lsmeans package (for zero‐inflated models) (Lenth, 2016). Path model was run using the piecewiseSEM package for R (Lefcheck, 2015).

## 3 RESULTS

### 3.1 Predator density

**Figure 1:**
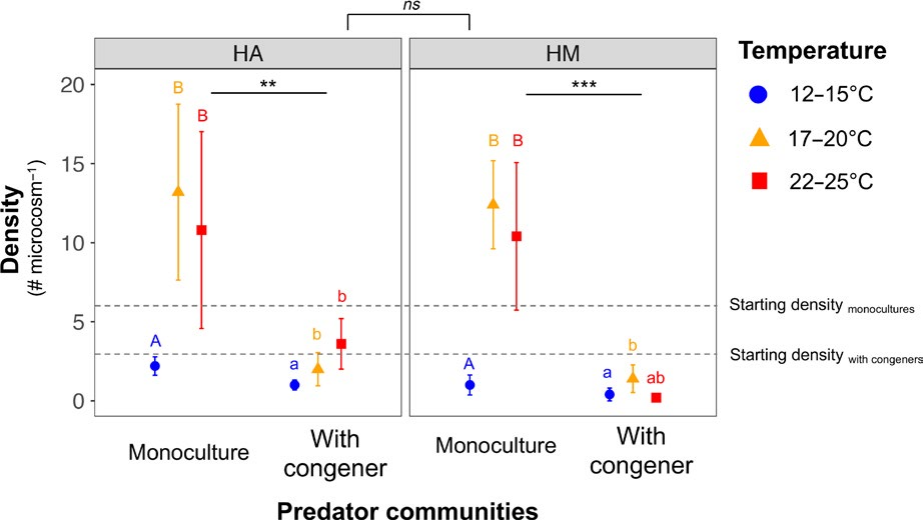
Changes in predator density in predator monocultures and predator polyculture across three temperature treatments at the end of the experiment. The values shown are mean density ± standard error. HA: *Hypoaspis aculeifer*, HM: *Hypoaspis miles*. The asterisk sign indicates the significant difference between the density of predators in their monoculture and when they were with congeners. The letters above the error bars are based on post‐hoc Tukey tests among the temperature treatments. The starting densities are indicated by the dashed lines. ns: not significant, **p‐value <0.01, ***p‐value <0.001

The final density of both predators significantly increased with temperature, whereas the presence of congeners had negative effects on their population (Figure 1, Table 1). The latter result is an indication of greater interspecific competition between the two congeneric predator species relative to intraspecific competition, and therefore, a negative effect of congeneric predator, although this was independent of warming (i.e., lack of significant interaction between temperature and predators, Table 1). The density of both predators was similar at the intermediate and high temperature in their monocultures, whereas *H. miles* showed a decline in the presence of *H. aculeifer* at the highest temperature (Figure 1). However, we did not find an overall difference between the density of these two predators when pooled over all treatments (*F*_1,54_ = 1.10, *p*‐value=0.30).

### 3.2 Prey density and traits

**Figure 2:**
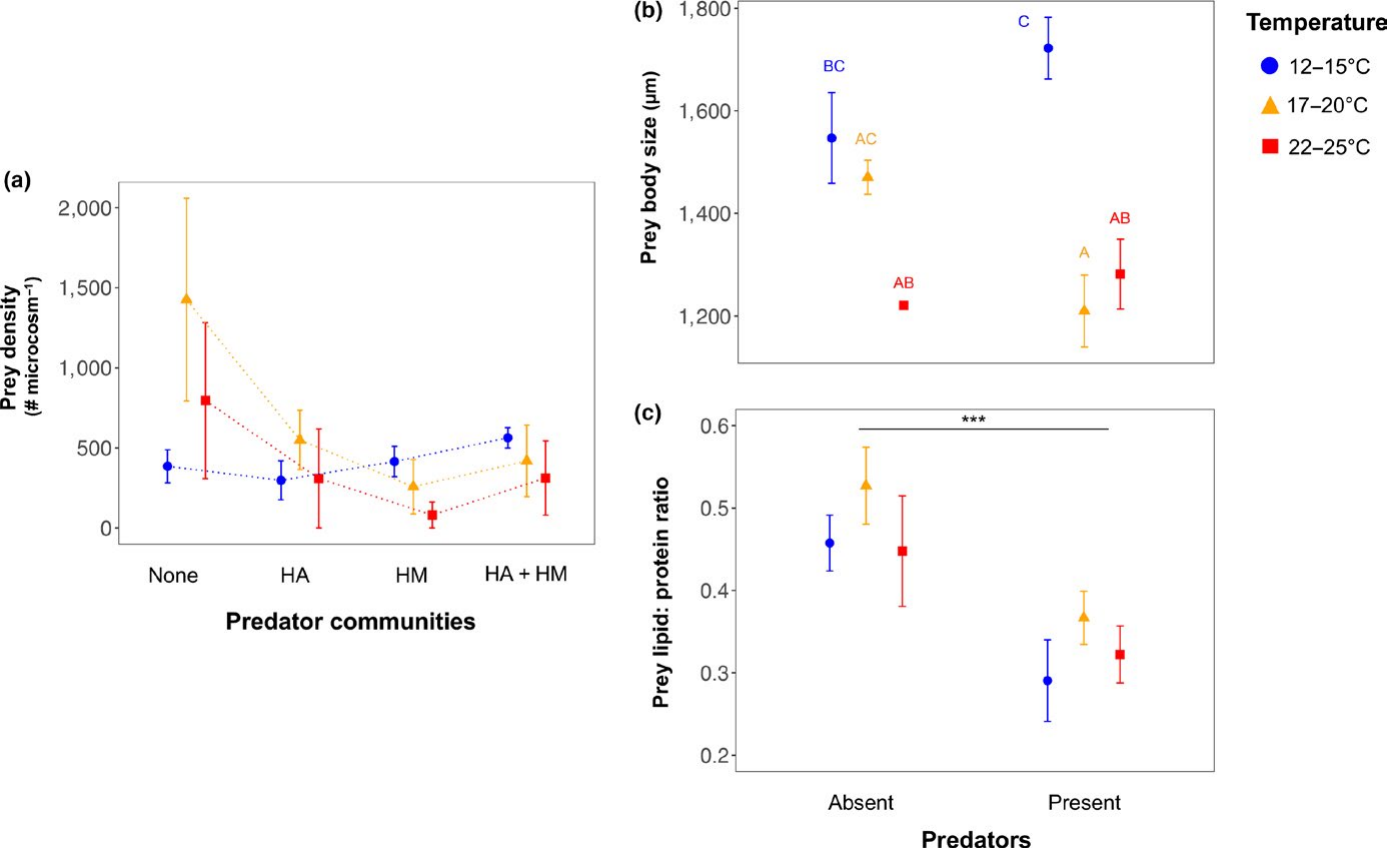
Prey responses to predator and temperature treatments. (a) Mean (± standard error) prey density in predator and temperature treatments at the end of the experiment. Here, prey density is the sum of the density of both prey species. Post‐hoc Tukey test showed no difference among groups. HA: *Hypoaspis aculeifer*, HM: *Hypoaspis miles*. (b) Mean (± standard error) prey body size (i.e., body length) in predator and temperature treatments. The prey body size is based on only *Folsomia candida* as the other prey (*Proisotoma minuta)* were insufficient in treatments with predator and higher temperature for the trait measurements. The letters above the error bars are based on post‐hoc Tukey tests. (c) Mean (± standard error) prey lipid: protein ratio in predator and temperature treatments. The lipid: protein ratio is also only of *F. candida* as the other prey (*P. minuta)* were insufficient in numbers in treatments with predator and higher temperature (Supporting Information Figure S2). Differences in predator effects are indicated by letters based on post‐hoc Tukey tests

The total prey density significantly decreased with temperature, but we found no significant effects of predator communities on prey density (Figure 2a, Table 1). Despite of significant temperature effects, we detected no statistical differences in group means among the three temperature treatments based on post‐hoc multiple comparisons. Interestingly, in the absence of predators, temperature seemed to increase prey density—a trend that disappeared in the presence of predators. However, we did not detect a significant interaction between predator communities and warming on the total density of prey. With respect to the two traits of surviving prey species (only of *F. candida*), we found a significant interaction between the presence of predators and warming on the body length of *F. candida* (Figure 2b, Table 1), whereas predators decreased the lipid: protein ratio of *F. candida* independent of warming (Figure 2c, Table 1). The body length of *F. candida* was higher at the ambient temperature in the presence of predators, but it declined notably at higher temperature irrespective of predators (Figure 2b).

### 3.3 Basal resources

**Figure 3:**
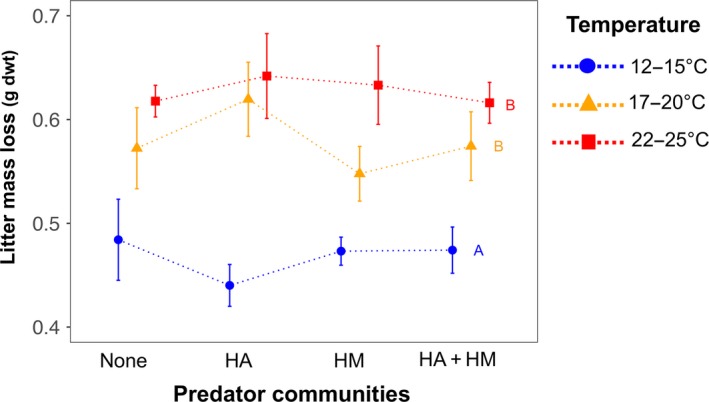
Greater effects of temperature on litter mass loss than predators. In the figure, we show mean (± standard error) litter mass loss in predator and temperature treatments. The letters above the error bars are based on post‐hoc Tukey tests among the temperature treatments. HA: *Hypoaspis aculeifer*, HM: *Hypoaspis miles*

We neither found any direct significant effect of temperature nor an effect of predator treatments on microbial biomass C (Table 1). In contrast, litter mass loss significantly increased at warmer temperatures (Figure 3, Table 1), but this effect was not modified by predators (Table 1). Moreover, litter mass loss was similar between the intermediate and high temperature treatments (Figure 3).

### 3.4 Path model

**Figure 4:**
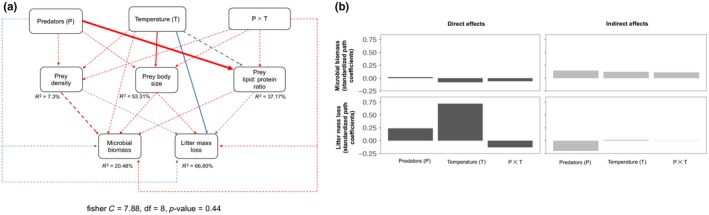
(a) Path model illustrating the direct and indirect effects of predators (presence and absence) and temperature on the two basal resources (Microbial biomass C, and litter biomass loss) via their effects on prey (*Folsomia candida*) density and traits. The sum of two species for total density is not used due to the availability of trait data only for *F. candida*. The thick lines are statistically significant paths, whereas dashed lines are statistically non‐significant. Blue arrows stand for positive path coefficients whereas red arrows indicate negative path coefficients. Arrow thickness is proportional to the respective (scaled) standardized coefficients (Supporting Information Table S2). (b) Total direct and indirect effects (based on the scaled standardized coefficients of path model) of predators and temperature on microbial biomass and litter mass loss. The calculation of indirect effects is explained in the method section. The magnitude of direct effects is listed in Supporting Information Table S2

Our path model fitted the data adequately as suggested by Fisher's C statistic (*p*‐value >0.05; Figure 4a). In the path model, only two paths were statistically significant (predator effects on the lipid: protein ratio of surviving prey and temperature effects on litter mass loss) and one was marginally significant (temperature effects on prey density; Figure 4a, Supporting Information Table S2). Further, we found greater direct effects of temperature on litter mass loss than predator effects, whereas we detected slightly greater indirect effects of predators on litter mass loss than temperature (Figure 4a,b). The indirect effect of predator presence on litter mass loss was negative compared to positive effects of temperature. The direct and indirect effects of temperature and predators on microbial biomass were minimal. The estimates for each path coefficient from Figure 4a are listed in Supporting Information Table S2.

## 4 DISCUSSION

As climate warming continues to alter the structure and function of ecosystems (Grimm et al., 2013; Traill, Lim, Sodhi, & Bradshaw, 2010; Wernberg et al., 2016), we need to enhance our understanding of the underlying mechanisms that render those alterations for improving our predictions of ecological communities in a warmer world (Gilman et al., 2010). In this study, we accordingly aimed to investigate whether warming alters the net effects of intra‐ and interspecific interactions at a higher trophic level (i.e., top predators) and whether those alterations have cascading effects on the lower trophic groups (i.e., prey and basal resources). Our experiment revealed greater interspecific competition (relative to intraspecific) between the two predators, but independent of temperature treatments. Temperature enhanced the density of predators in their monocultures. Shifts in prey traits were driven by both predators and temperature, although prey density declined only in warmer treatments. Opposite trends in the density of predators and prey in warmer environments indicate a trophic mismatch in the experimental community of this study. Further, we did not detect any interactive effect of temperature and predators on the two basal resources (Table 1). Temperature effects on litter loss were much stronger than predators. Our results thus indicate weaker predator effects on lower trophic groups than that of temperature.

One of the main results of our study is negative interactions between the two predatory mite species independent of temperature treatments (i.e., lack of interaction effect between temperature and predator communities on predator density; Figure 1, Table 1). Negative interactions between two predators persisted despite of overall increase in their density at higher temperatures (Table 1). The temperature‐induced increase in predator density corresponds to several laboratory studies on predatory mites (Lobbes & Schotten, 1980; Wright & Chambers, 1994), which is often due to accelerated life cycle at higher temperature in resource‐rich environments. The negative interactions between the two predator species are likely to relate to their exploitative and interference competitive interactions particularly due to their high taxonomic relatedness (Griffin et al., 2013). As temperature did not reduce the resources to an alarming level as confirmed by prey population and the greater predator density at the higher temperature (in their monocultures; Figures 1 and 2a), we could speculate that interspecific competition between the predators was pronounced mainly due to elevated interference than exploitative competition.

The temperature‐independent interspecific competition (relative to intraspecific competition) between the two predator species is surprising as temperature would usually increase consumption rates (Amarasekare, 2015) that require greater movement among the individuals and thus a greater likelihood of interference (Lang et al., 2012). This could probably be due to weaker effects of abiotic stress (temperature in this case) on communities when interspecific interactions (biotic interactions) among the member species are strong (Post, 2013). Moreover, predatory mites could feed on the eggs of conspecifics giving rise to intraguild predation (Walzer & Schausberger, 1999), which may also have played some role in the observed density patterns of predators in mixed predator communities. Although intraguild predation is usually less evident when species are of similar body size and larger spatial overlap for the shared prey compared to interference (Schmitz, 2007), predatory mites are highly active foragers, which often leads to intraguild predation (Start & Gilbert, 2017). Predator effects were mainly observed for prey lipid: protein ratio (Figure 2c, Table 1). A decline in lipid: protein ratio of the prey species shows constraints for maximizing their energy uptake in the presence of predators. Consumers (prey species *F. candida* in this case) usually maximize lipid: protein ratio for maximizing energy uptake from the available food (fungi and litter in this study) due to greater caloric content of lipids than proteins (Jensen et al., 2012). This is in accordance with predator‐induced physiological stress in prey individuals (Hawlena & Schmitz, 2010), which may lead to their dietary shifts (Winnie & Creel, 2017).

Temperature effects on the prey species were observed for both density and body size. A significant decline of prey density at higher temperature is indicative of increased thermal stress for most of the prey individuals, although particularly at the highest temperature (Figure 2a). Thus, temperature triggered contrasting responses between prey species (namely *F. candida*) and the two predators. This result points out that prey species that are larger than their predators are likely to be more vulnerable to warming than their predators, which agrees with the notion of metabolic mismatches between trophic groups (Iles, 2014; Rall, Vucic‐Pestic, Ehnes, Emmerson, & Brose, 2010). However, in the long run positive effects of temperature on predators are bound to disappear owing to starvation caused by lower prey availability. Further, the reduction in the size of the prey species (of *F. candida)* at higher temperatures corresponded well with the widely claimed phenomenon of organismal size reduction in response to warming (Gardner et al., 2011; Sheridan & Bickford, 2011). Such a decline in the body size relates to organisms’ optimization of metabolic efficiency at higher temperature (Gardner et al., 2011; Gunderson & Leal, 2016). Interestingly, our finding of greater body size of *F. candida* in the presence of predators at ambient temperature suggests that ectotherms may face a trade‐off between growing large to offset predation and shrinking in size to offset metabolic costs at higher temperature. In this study, the latter seemed to be the case when the prey species were exposed to higher temperature and predators.

The direct effect of temperature was dominant in driving the litter mass loss, whereas predator presence showed relatively stronger indirect effects on the same (Figure 4b). The strength of direct effects of temperature was remarkably larger than the indirect effects of predators on this basal resource. This, in part, is potentially owing to weaker effects of predators on prey density and trait compared to multitrophic direct effects of warming (Table 1). Such a result has important implications for understanding how warming is going to reinforce changes in food web structure even when their effects are moderate at higher trophic levels. We may expect such scenarios particularly where prey is relatively larger than the predators in food webs and thus are more responsive to warming as was the case in our study.

In summary, our results indicate that temperature effects on prey and basal resources were stronger than that of predators, despite greater predator density at higher temperature and interspecific (relative to intraspecific) competition between the two predators. These results further suggest that temperature sensitivity at different trophic levels may vary (Dell, Pawar, & Savage, 2011) and likely depend on the context of biotic interactions, which are crucial in predicting trophic cascades in complex communities in warmer environments. For instance, the cascading effect of (temperature‐independent) interspecific interactions between the two congeneric predators was seemingly weak in our study. We show that temperature sensitivity of lower trophic groups was higher than the effects of higher trophic groups, implying that warmer environments can also alter ecosystem functions without altering interactions at the higher trophic levels. By no means do we claim here to discount the crucial roles of intra‐ and interspecific interactions among higher trophic species in shaping ecosystem structure and function (Estes et al., 2011; Schmitz, Hawlena, & Trussell, 2010). Our own results confirm that predatory interactions shifted the diet of a prey species as shown by their lipid: protein ratio, which may have several implications, such as for the diversity of basal resources that we did not measure in our study. Nevertheless, we highlight that effects of climate warming can exceed that of predator effects on the amount of basal resources potentially via their greater effects on prey population size, traits and direct effects on a basal resource.

## CONFLICT OF INTEREST

None declared.

## AUTHOR CONTRIBUTIONS

MPT conceived the study. TK and MPT performed the experiment. NE provided the facilities to perform the experiment. TK, MPT, SD, and AF collected the data. MPT analyzed the data and wrote the manuscript with substantial contributions from JNG and NE. All coauthors contributed to revisions.

## DATA ACCESSIBILITY

We agree to deposit the data used in this study in Dryad (https://doi.org/10.5061/dryad.4d15b08).
