# Supplementary material for: Temperature effects on prey and basal resources exceed that of predators in an experimental community
Source: Ecol Evol. 2018 Nov 26;8(24):12670–80. doi: 10.1002/ece3.4695 (PMC6308891; doi:10.1002/ece3.4695)
Supplement: Supplementary file 1 [file ECE3-8-12670-s001.docx]

**Supplementary information**

**Temperature effects on prey and basal resources exceed that of predators in an experimental community**

Madhav P. Thakur^1,2,3^, John N. Griffin^4^, Tom Künne^1,2^, Susanne Dunker^1,5^, Andrea Fanesi^2^, Nico Eisenhauer^1,2^

*^1^ German Centre for Integrative Biodiversity Research (iDiv) Halle-Jena-Leipzig, Deutscher Platz 5e, 04103 Leipzig, Germany*

*^2^ Institute of Biology, Leipzig University, Deutscher Platz 5e, 04103 Leipzig, Germany*

*^3^* *Netherlands Institute of Ecology (NIOO-KNAW), 6700 AB Wageningen, The Netherlands*

*^4^ Department of Biosciences, Swansea University, Wallace Building, Singleton Park, Swansea, SA2 8PP, UK*

*^5^ Helmholtz Centre for Environmental Research – UFZ, Leipzig, Germany*

Correspondence: Madhav P. Thakur; email: [madhav.prakash.thakur@gmail.com](mailto:madhav.prakash.thakur@gmail.com); Telephone: +31 (0)317 473 416


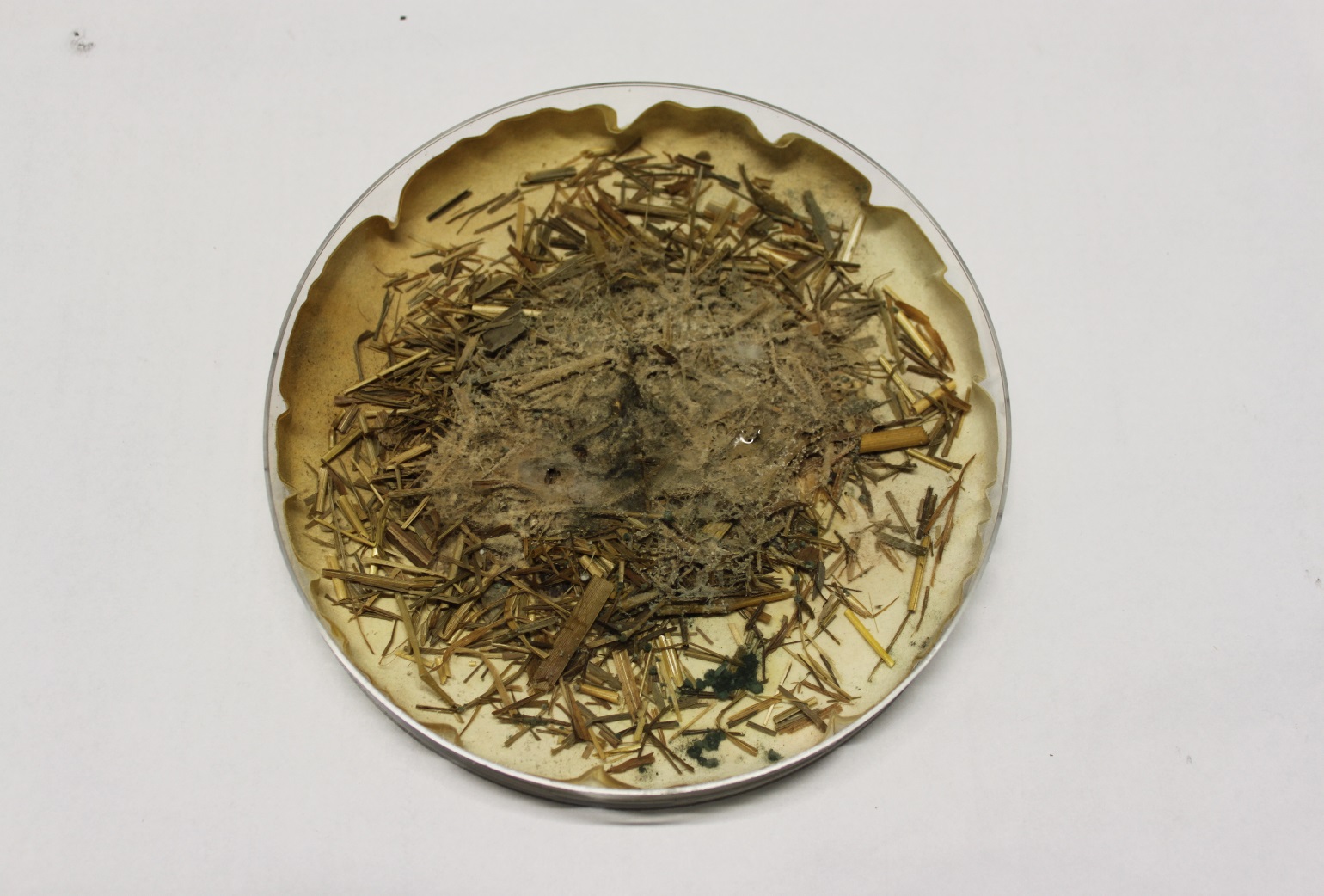

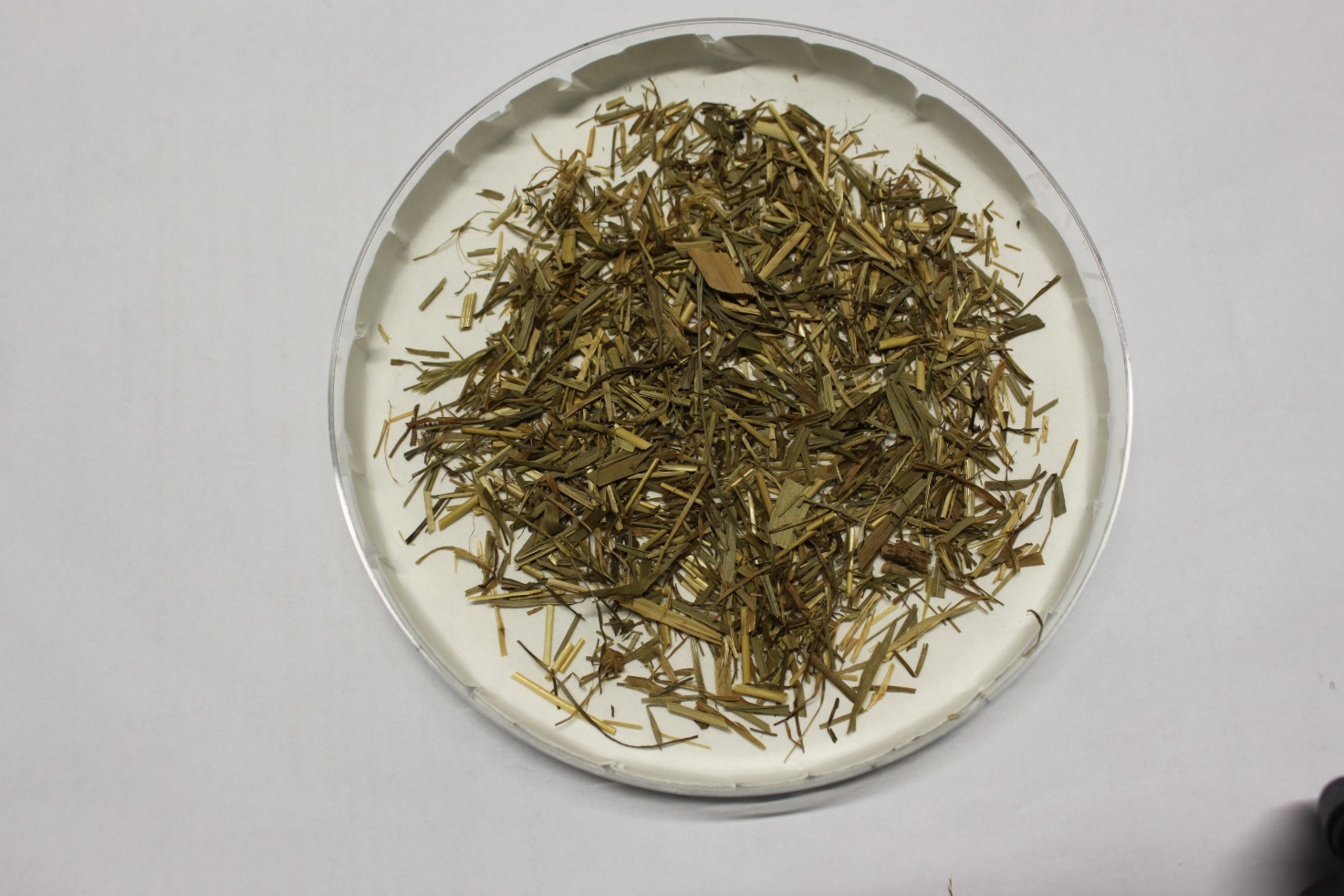


**Figure S1: (Left)** The microcosm (petri-dish) with litter as the substrate (without yeast and moisture) before the start of the experiment. We used a double layer milk filter to retain moisture for a longer period. **(Right)** The microcosm in the final week of the experiment.

**Figure S2:** Density of *Proisotoma minuta* at the end of the experiment in different treatments. The individuals of *P. minuta* were often lower than 10 in presence of predators and higher temperature, which prevented us to measure their traits.

**Figure S3:** Histogram depicting zero-inflation in prey density data (disproportionally higher number of zero counts)

**Table S1:** Results of linear mixed models with temperature and predator richness effects on predator and prey densities, prey traits and basal resources. The statistical significance (F- and p-values) of the response variable was determined by F-tests. P-values less than 0.05 are statistically significant and indicated in bold. df stands for degree of freedom.

|  |  |  |  |  |  |  |  |  |  |  |  |  |  |
| --- | --- | --- | --- | --- | --- | --- | --- | --- | --- | --- | --- | --- | --- |
|  |  |  |  |  |  |  |  |  |  |  |  |  |  |
|  |  | **Predator communities (P)** | | | | **Temperature (T)** | | | | **P🞨T** | | |  |
|  |  | Slope (confidence interval) | F-value_df_ | p-value |  | Slope (confidence interval) | F-value_df_ | p-value |  | Slope (confidence interval) | F-value_df_ | p-value |  |
|  | **Predator response** | | | | | | | | | | | |  |
|  | *H. aculeifer* density | -1.27 (-2.88, 0.29) | **11.65_1,26_** | **<0.01** |  | 0.86 (-1.06, 2.82) | **6.48_1,26_** | **0.01** |  | -0.11 (-1.32, 1.10) | 0.04_1,26_ | 0.83 |  |
|  | *H. miles* density | -0.97 (-3.20, 1.22) | **15.40_1,26_** | **<0.001** |  | 2.37 (-0.36, 5.24) | 1.58_1,26_ | 0.21 |  | -1.29 (-3.19, 0.52) | 2.31_1,26_ | 0.14 |  |
|  | **Prey response** | | | | | | | | | | | |  |
|  | Prey density | 0.18 (-0.50, 0.35) | **4.94_1,54_** | **0.03** |  | 1.28 (-1.38, 0.25) | **6.02_1,54_** | **0.01** |  | 0.17(0.12, 0.96) | **6.54_1,54_** | **0.01** |  |
|  | Body size | 92.38 (-110.94, 295.71) | <0.01_1,34_ | 0.98 |  | -152.66 (-314.58, 9.26) | **36.94_1,34_** | **<0.001** |  | 123.61(-311.24, 64.02) | 1.79_1,34_ | 0.18 |  |
|  | Lipid:protein ratio | -0.17 (-0.28, -0.06) | **16.40_1,31_** | **<0.001** |  | <0.01 (-0.08, 0.08) | 0.98_1,31_ | 0.60 |  | 0.02(-0.07, 0.12) | 0.26_1,31_ | 0.6 |  |
|  | **Basal resource response** | | | | | | | | | | | |  |
|  | Microbial biomass C (log-transformed) | -0.03 (-0.26, 0.18) | 0.60_1,54_ | 0.43 |  | -0.11 (-0.32, 0.08) | 0.29_1,54_ | 0.59 |  | 0.08 (-0.08, 0.25) | 1.07_1,54_ | 0.30 |  |
|  | Litter mass loss | -0.004 (-0.07, 0.06) | <0.01_1,56_ | 0.94 |  | 0.14 (0.07, 0.20) | **61.97_1,56_** | **<0.001** |  | 0.002(-0.05, 0.05) | <0.01_1,56_ | 0.92 |  |
|  |  |  |  |  |  |  |  |  |  |  |  |  |  |

**Table S2:** Results of path model analyses on the effects of predators and temperature on the two basal resources via prey density and traits. Estimates are scaled standardized coefficients from the path model. Bold values are statistically significant (p-value <0.05).

|  |  |  |  |  |  |  |
| --- | --- | --- | --- | --- | --- | --- |
|  | **Predictor** | **Response variables** | **Estimate** | **Std. error** | **p-value** |  |
|  | Predators (P) | Prey density | -0.14 | 0.33 | 0.65 |  |
|  | Temperature (T) | Prey density | -0.17 | 0.28 | 0.53 |  |
|  | P * T | Prey density | -0.23 | 0.33 | 0.48 |  |
|  | P | Prey body size | -0.11 | 0.28 | 0.68 |  |
|  | T | Prey body size | *-0.47* | *0.24* | *0.06* |  |
|  | P * T | Prey body size | -0.11 | 0.28 | 0.18 |  |
|  | P | Prey lipid:protein ratio | **-1.24** | **0.33** | **<0.01** |  |
|  | T | Prey lipid:protein ratio | 0.02 | 0.28 | 0.92 |  |
|  | P * T | Prey lipid:protein ratio | 0.17 | 0.34 | 0.60 |  |
|  | P | Microbial biomass | 0.02 | 0.57 | 0.96 |  |
|  | T | Microbial biomass | -0.08 | 0.48 | 0.86 |  |
|  | P * T | Microbial biomass | -0.06 | 0.46 | 0.88 |  |
|  | P | Litter mass loss | 0.24 | 0.31 | 0.44 |  |
|  | T | Litter mass loss | **0.72** | **0.27** | **0.01** |  |
|  | P * T | Litter mass loss | -0.13 | 0.26 | 0.60 |  |
|  | Prey density | Microbial biomass | -0.49 | 0.32 | 0.14 |  |
|  | Prey body size | Microbial biomass | -0.09 | 0.30 | 0.74 |  |
|  | Prey lipid:protein ratio | Microbial biomass | -0.06 | 0.26 | 0.80 |  |
|  | Prey density | Litter mass loss | 0.17 | 0.18 | 0.35 |  |
|  | Prey body size | Litter mass loss | -0.09 | 0.17 | 0.58 |  |
|  | Prey lipid:protein ratio | Litter mass loss | 0.15 | 0.13 | 0.24 |  |
|  |  |  |  |  |  |  |
